# The influence of atrial fibrillation on the levels of NT-proBNP versus GDF-15 in patients with heart failure

**DOI:** 10.1007/s00392-019-01513-y

**Published:** 2019-07-01

**Authors:** Bernadet T. Santema, Michelle M. Y. Chan, Jasper Tromp, Martin Dokter, Haye H. van der Wal, Johanna E. Emmens, Janny Takens, Nilesh J. Samani, Leong L. Ng, Chim C. Lang, Peter van der Meer, Jozine M. ter Maaten, Kevin Damman, Kenneth Dickstein, John G. Cleland, Faiez Zannad, Stefan D. Anker, Marco Metra, Pim van der Harst, Rudolf A. de Boer, Dirk J. van Veldhuisen, Michiel Rienstra, Carolyn S. P. Lam, Adriaan A. Voors

**Affiliations:** 1 Department of Cardiology, University of Groningen, University Medical Centre Groningen, Hanzeplein 1, 9713 GZ Groningen, The Netherlands; 2 Department of Cardiology, National Heart Centre Singapore, Singapore Duke-NUS Graduate Medical School, Singapore, Singapore; 3 Department of Cardiovascular Sciences, University of Leicester, Groby Road, Leicester, LE3 9QP UK; 4 NIHR Leicester Biomedical Research Centre, Glenfield Hospital, Groby Road, Leicester, LE3 9QP UK; 5 Division of Molecular and Clinical Medicine, School of Medicine University of Dundee, Ninewells Hospital and Medical School, Dundee, DD1 9SY UK; 6 University of Bergen, Bergen, Norway; 7 Stavanger University Hospital, Stavanger, Norway; 8 National Heart & Lung Institute, Royal Brompton & Harefield Hospitals, Imperial College, Sydney St, Chelsea, London, SW3 6NP UK; 9 Robertson Institute of Biostatistics and Clinical Trials Unit, University of Glasgow, University Avenue, Glasgow, G12 8QQ UK; 10 Inserm CIC 1433, Université de Lorrain, CHU de Nancy, Nancy, France; 11 Department of Cardiology (CVK), and Berlin Institute of Health Center for Regenerative Therapies (BCRT), German Centre for Cardiovascular Research (DZHK) partner site Berlin, Charité Universitätsmedizin, Berlin, Germany; 12 Department of Medical and Surgical Specialties, Radiological Sciences and Public Health, Institute of Cardiology, University of Brescia, Brescia, Italy

**Keywords:** Atrial fibrillation, Heart failure, Biomarkers, Natriuretic peptides, GDF-15

## Abstract

**Background:**

In heart failure (HF), levels of NT-proBNP are influenced by the presence of concomitant atrial fibrillation (AF), making it difficult to distinguish between HF versus AF in patients with raised NT-proBNP. It is unknown whether levels of GDF-15 are also influenced by AF in patients with HF. In this study we compared the plasma levels of NT-proBNP versus GDF-15 in patients with HF in AF versus sinus rhythm (SR).

**Methods:**

In a post hoc analysis of the index cohort of BIOSTAT-CHF (*n* = 2516), we studied patients with HF categorized into three groups: (1) AF at baseline (*n* = 733), (2) SR at baseline with a history of AF (*n* = 183), and (3) SR at baseline and no history of AF (*n* = 1025). The findings were validated in the validation cohort of BIOSTAT-CHF (*n* = 1738).

**Results:**

Plasma NT-proBNP levels of patients who had AF at baseline were higher than those of patients in SR (both with and without a history of AF), even after multivariable adjustment (3417 [25th–75th percentile 1897–6486] versus 1788 [682–3870], adjusted *p* < 0.001, versus 2231 pg/mL [902–5270], adjusted *p* < 0.001). In contrast, after adjusting for clinical confounders, the levels of GDF-15 were comparable between the three groups (3179 [2062–5253] versus 2545 [1686–4337], adjusted *p* = 0.36, versus 2294 [1471–3855] pg/mL, adjusted *p* = 0.08). Similar patterns of both NT-proBNP and GDF-15 were found in the validation cohort.

**Conclusion:**

These data show that in patients with HF, NT-proBNP is significantly influenced by underlying AF at time of measurement and not by previous episodes of AF, whereas the levels of GDF-15 are not influenced by the presence of AF. Therefore, GDF-15 might have additive value combined with NT-proBNP in the assessment of patients with HF and concomitant AF.

**Graphic abstract:**

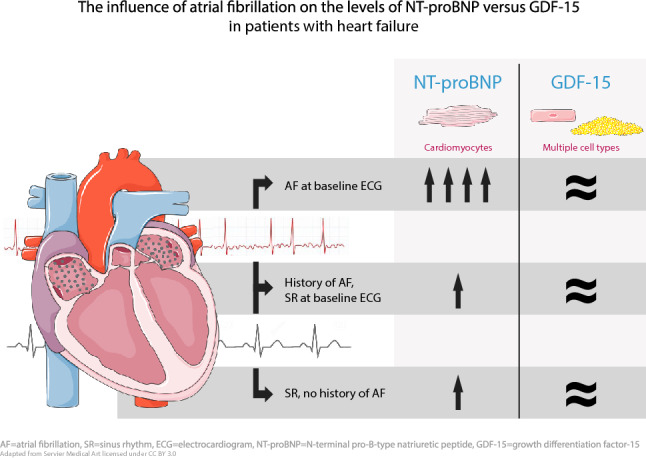

**Electronic supplementary material:**

The online version of this article (10.1007/s00392-019-01513-y) contains supplementary material, which is available to authorized users.

## Introduction

N-terminal pro-B-type natriuretic peptide (NT-proBNP) plays an important role in the diagnosis and prognosis of heart failure (HF) [1, 2]. For the diagnosis of HF, this marker is known for its high sensitivity, but lower specificity, which makes NT-proBNP especially helpful to rule out HF [3]. Several other conditions, such as renal failure, pulmonary embolism and atrial fibrillation (AF), are also known to further elevate NT-proBNP levels in patients with concomitant HF. AF in this regards is particularly important because it is highly prevalent among patients with HF regardless of ejection fraction, and mimics the symptoms (breathlessness) and signs (left atrial enlargement) of HF. Therefore, it is often unclear how elevated NT-proBNP levels in patients with HF and AF should be interpreted, since there are several potential explanations for these elevated levels [4–6]. First, NT-proBNP elevations may be directly related to the immediate hemodynamic alterations during the actual episode of AF [7]. Secondly, elevated levels of NT-proBNP may be related to the chronic structural or functional cardiac remodeling as a result of sustained episodes of AF, or in the third place, just reflect that patients with AF have more advanced HF. In most contemporary clinical HF trials, different NT-proBNP thresholds are being used for inclusion of patients with and without AF, often without further differentiation between patients who only have a history of AF, and those who have AF at time of enrollment.

In the past years, many markers have been shown to have strong prognostic value in HF, of which growth differentiation factor-15 (GDF-15) is amongst the best established ones [8–11]. GDF-15 is a protein belonging to the transforming growth factor-beta superfamily, and has a role in inflammatory and apoptotic cell processes, and is produced by multiple organs, including the heart [12–14]. It is, however, unknown how the plasma levels of GDF-15 are influenced by underlying AF in patients with HF. The search for a biomarker that is less influenced by underlying AF than NT-proBNP is, could be of help in the assessment of patients with both HF and AF.

To investigate whether the levels of GDF-15 are similarly elevated as NT-proBNP by concomitant AF in patients with HF, we performed a post hoc analysis of these two biomarkers in The BIOlogy Study to Tailored Treatment in Chronic Heart Failure (BIOSTAT-CHF) [15].

## Methods

### Patient population and definitions

In the multinational, prospective, observational index cohort of BIOSTAT-CHF, 2516 patients with new-onset or worsening signs and/or symptoms of HF from 11 European countries were included between 2010 and 2012 [15]. Patients had to have evidence of cardiac dysfunction documented either by left ventricular ejection fraction (LVEF) of ≤ 40% or plasma concentrations of NT-proBNP > 2000 pg/mL (this cutoff was the same for patients in sinus rhythm [SR] and AF). A comparable validation cohort of BIOSTAT-CHF included another 1738 patients from six centers in Scotland between 2010 and 2014, who had to have a previously documented admission for HF. No additional LVEF or NT-proBNP was used for the validation cohort, which resulted in a higher percentage of patients with heart failure with preserved ejection fraction (HFpEF, 34% in the validation cohort versus 7% in the index cohort).

In both cohorts, a standard 12-lead electrocardiogram (ECG) was performed at baseline, generally on the same day as the time of blood draw for biomarker measurements (median difference of 0 days with 25th–75th percentile [Q1–Q3] from − 2 to + 2 days. Patients were categorized into three groups based on history and baseline ECG: (1) AF at baseline, (2) history of AF but in SR at baseline, and (3) SR at baseline and no previously documented episode of AF. Patients with a rhythm other than SR or AF on the baseline ECG were excluded (pacemaker rhythm, *n* = 283, and unknown rhythm, *n* = 55) [16]. The study was conducted according to the Declaration of Helsinki, approved by the medical ethics committees of participating centers, and all patients provided informed consent.

### Biomarkers

Measurement of NT-proBNP and GDF-15 was performed at baseline. The levels of NT-proBNP and GDF-15 were measured using electrochemiluminescence on a cobas e 411 analyzer, using standard methods (Roche Diagnostics GmbH, Mannheim, Germany) [17, 18].

### Statistical analyses

Normally distributed variables were displayed as mean with standard deviation, non-normally distributed variables as median with 25th–75th percentile, and categorical variables as numbers with percentages. Group differences were assessed with *t tests* and one-way analysis of variance for normally distributed variables, Kruskal–Wallis and Mann–Whitney *U* tests for non-normally distributed continuous variables, and *χ*^2^ tests for categorical variables. Multiple linear regression models were used to investigate the associations between NT-proBNP and GDF-15 and the three rhythm groups. Natural transformed biomarkers were used in the regression analyses. Potential and known confounders of the two biomarkers were included in the regression model, including age, sex, body mass index (BMI), LVEF, heart rate, renal disease [estimated glomerular filtration rate (eGFR) using the Chronic Kidney Disease Epidemiology Collaboration (CKD-EPI) formula], a previous myocardial infarction, diabetes mellitus, and the use of ACE-inhibitors/ARBs and beta-blockers at baseline. To test which variables had the strongest association with elevated levels of NT-proBNP and GDF-15, both biomarkers were analyzed as a dependent variable in uni- and multi-variable linear regression analysis. The multivariable models were built with all variables with *p* < 0.10 in the univariable analysis, after which backward elimination was performed. Variables that had the strongest associations in both the index and validation cohort were displayed in the final multivariable model. A *p* value of < 0.1 was considered significant for testing interactions. *p* values of < 0.05 were considered statistically significant in all other analyses. All analyses were conducted with R version 3.5.2 (R Foundation for Statistical Computing, Vienna, Austria).

## Results

### Index cohort

A total of 1941 patients with HF were studied, of whom 733 patients had AF at baseline, 183 patients had a history of AF but were in SR at baseline, and 1025 had SR at baseline and no previously documented episode of AF. The characteristics of the patients within these three rhythm groups are summarized in Table 1. Main findings were that patients with AF at baseline were significantly older, had a higher BMI and higher heart rate, and less often had a previous myocardial infarction as compared with the two other groups who were in SR at baseline.

**Table 1:** Baseline characteristics of the index cohort, stratified by heart rhythm

Clinical characteristic	AF at baseline *N* = 733 (38%)	History of AF–SR at baseline *N* = 183 (9%)	Sinus rhythm *N* = 1025 (53%)	*P* for trend
Age (years)	76 ± 10	72 ± 11	70 ± 13	< 0.001
Women (%)	182 (25)	51 (28)	301 (29)	0.110
BMI (kg/m^2^)	28.5 ± 5.6	28.3 ± 5.1	27.5 ± 5.5	0.001
NYHA (%)				0.010
I/II	202 (28)	68 (37)	331 (32)	
III	233 (36)	37 (23)	270 (31)	
IV	28 (4)	7 (4)	33 (4)	
LVEF, %	33 ± 12	33 ± 11	29 ± 10	< 0.001
Systolic blood pressure (mmHg)	125 ± 22	126 ± 24	126 ± 22	0.780
Diastolic blood pressure (mmHg)	76 ± 14	75 ± 16	75 ± 13	0.188
Heart rate (beats/min)	93 ± 25	73 ± 16	79 ± 18	< 0.001
History of (%)				
Myocardial infarction	215 (29)	67 (37)	431 (42)	< 0.001
Stroke	83 (11)	27 (15)	72 (7)	< 0.001
Hypertension	470 (64)	118 (65)	623 (61)	0.300
Diabetes mellitus	232 (32)	63 (34)	320 (31)	0.691
COPD	133 (18)	46 (25)	150 (15)	0.001
Medication (%)				
ACE-inhibitors/ARBs	504 (69)	135 (74)	770 (75)	0.012
Beta-blockers	599 (82)	160 (87)	850 (83)	0.185
Loop diuretics	732 (100)	183 (100)	1024 (100)	0.873
Amiodarone	92 (13)	67 (37)	117 (11)	< 0.001
Digoxin	282 (39)	21 (12)	72 (7)	< 0.001
Verapamil/diltiazem	18 (3)	4 (2)	7 (1)	0.008
Class 1c antiarrhythmic drugs	2 (1)	5 (3)	2 (1)	< 0.001
Ivabradine	0 (0)	2 (1)	26 (3)	< 0.001
Laboratory data				
eGFR	58.0 ± 21.8	59.2 ± 20.8	66.0 ± 23.4	< 0.001
NT-proBNP (pg/mL)	3417 [1897, 6486]	1788 [682, 3870]	2231 [902, 5270]	< 0.001
GDF-15 (pg/mL)	3197 [2062, 5253]	2545 [1686, 4337]	2294 [1471, 3855]	< 0.001

*AF* atrial fibrillation, *SR* sinus rhythm, *BMI* body mass index, *NYHA* New York Heart Association, *LVEF* left ventricular ejection fraction, *COPD* chronic obstructive pulmonary disease, *ACE* angiotensin converting enzyme, *ARB* angiotensin receptor blockers, *eGFR* estimated glomerular filtration rate, *NT*-*proBNP* N-terminal pro-B-type natriuretic peptide, *GDF15* growth differentiation factor 15

The plasma levels of NT-proBNP were significantly higher in patients who had AF at baseline, with a median of 3417 pg/mL (1897–6486), as compared with patients who were in SR at baseline; both those who had a history of AF (1788 pg/mL [682–3870], *p* < 0.001) and those who never had AF before (1588 pg/mL [902–5270], *p* < 0.001) (Table 1), also after multivariable adjustment (Table 2). In univariable analysis, the levels of GDF-15 were also highest in patients with AF at baseline and lowest in patients who were in SR at baseline (Table 1), but after adjusting for clinical confounders, the levels of GDF-15 between patients with AF at baseline and those in SR with and without previous AF were comparable (Table 2).

**Table 2:**
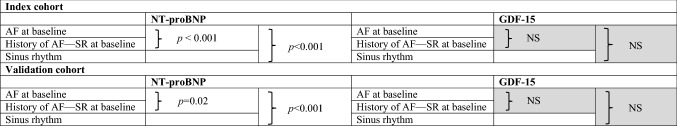
Multivariable differences of the plasma levels of NT-proBNP and GDF-15 between the three rhythm groups in the index and validation cohort

Adjusted for age, sex, body mass index, left ventricular ejection fraction, heart rate, estimated glomerular filtration rate, a previous myocardial infarction, diabetes mellitus, and the use of ACE-inhibitors/ARBs and beta-blockers at baseline

*NT-proBNP* N-terminal pro-B-type natriuretic peptide, *GDF15* growth differentiation factor 15, *NYHA* New York Heart Association, *BMI* body mass index, *LVEF* left ventricular ejection fraction, *eGFR* estimated glomerular filtration rate, *NS* non-significant

### Validation cohort

Baseline characteristics of the validation cohort were generally comparable to the index cohort of BIOSTAT-CHF (Supplementary Table 1). As discussed previously, a higher number of patients with HFpEF were included in the validation cohort, which resulted in a higher number of women, a higher LVEF, and lower levels of NT-proBNP in all three groups. Despite these differences in baseline characteristics, similar patterns of plasma levels of both NT-proBNP and GDF-15 were found in the validation cohort as compared to the index cohort. Patients with AF at baseline had a median NT-proBNP of 2105 pg/mL (1015–4472), which was significantly higher than those who had a history of AF but were in SR at baseline (1063 [440–4094], *p* < 0.001) and patients in SR who never had AF before (874 [314–2758]). The levels of GDF-15 were comparable among the three groups after multivariable adjustment (Table 2). No significant interactions between the biomarkers and the rhythm groups in heart failure with reduced ejection fraction (HFrEF) versus HFpEF were found in both the index and validation cohort.

### Correlates of NT-proBNP versus GDF-15

The multivariable models with the correlates of elevated levels of NT-proBNP and GDF-15 are presented in Table 3. AF at baseline was strongly associated with elevated levels of NT-proBNP in both the index and validation cohort. Other variables that were strongly associated with elevated levels of NT-proBNP were age, BMI, LVEF and eGFR. AF at baseline was not associated with higher levels of GDF-15 in the multivariable model. Variables that were strongly associated with higher levels of GDF-15 in both cohorts were age, systolic blood pressure, diabetes mellitus and eGFR.

**Table 3:** Multivariable model with the strongest associated correlates of NT-proBNP and GDF-15 in the index and validation cohort

Variable	NT-proBNP						GDF-15					
	Index cohort			Validation cohort			Index cohort			Validation cohort		
	Beta Coeff	*T* value	*P* value	Beta Coeff	*T* value	*P* value	Beta Coeff	*T* value	*P* value	Beta Coeff	*T* value	*P* value
Age	0.014	3.291	0.001	0.021	3.613	< 0.0001	0.009	4.033	< 0.0001	0.010	3.605	< 0.0001
BMI	− 0.071	− 8.846	< 0.0001	− 0.076	− 9.015	< 0.0001	NS	NS	NS	NS	NS	NS
Systolic blood pressure	NS	NS	NS	NS	NS	NS	− 0.005	− 3.972	< 0.0001	− 0.004	− 3.244	0.001
LVEF	− 0.022	− 5.232	< 0.0001	− 0.028	− 7.402	< 0.0001	NS	NS	NS	NS	NS	NS
Atrial fibrillation at baseline	0.438	4.550	< 0.0001	0.766	7.163	< 0.0001	NS	NS	NS	NS	NS	NS
Diabetes mellitus	NS	NS	NS	NS	NS	NS	0.412	7.543	< 0.0001	0.291	5.461	< 0.0001
Heart rate	0.017	8.004	< 0.0001	0.016	6.669	< 0.0001	0.007	5.620	< 0.0001	0.006	5.510	< 0.0001
eGFR	− 0.022	− 10.469	< 0.0001	− 0.023	− 9.070	< 0.0001	0–0.02	− 12.952	< 0.0001	− 0.019	− 14.294	< 0.0001
NYHA class IV	0.821	3.343	0.0008	1.597	3.168	0.002	0.332	2.239	0.025	0.972	3.770	< 0.0001
	*r*^2^ = 0.261			*r*^2^ = 0.353			*r*^2^ = 0.244			*r*^2^ = 0.335		

*NT*-*proBNP* N-terminal pro-B-type natriuretic peptide, *GDF15* growth differentiation factor 15, *NYHA* New York Heart Association, *BMI* body mass index, *LVEF* left ventricular ejection fraction, *eGFR* estimated glomerular filtration rate, *NS* non-significant

## Discussion

These data suggest that in patients with HF, after adjustment for clinical confounders, the levels of NT-proBNP, but not GDF-15, are significantly influenced by the presence of AF at time of measurement. GDF-15 is mainly produced in non-cardiac and peripheral tissues, such as endothelial cells and adipocytes, and we recently showed that the levels of GDF-15 in mice are 2- to 60-fold higher in the liver, lungs and kidney than in the cardiac muscle [19]. Therefore, this marker reflects changes in many organs, not just in the cardiac ventricles and atria, and might therefore be less likely to be load-dependent as compared with NT-proBNP. Since levels of GDF-15 are independent of the presence of AF in patients with HF, it may better reflect HF patients’ overall clinical condition, including non-cardiac comorbidities [20].

Previous studies have shown that the levels of GDF-15 in AF patients without HF are fairly similar to the levels in community-dwelling elderly [21]. In the AF field, GDF-15 is of increasing interest since this biomarker was the strongest predictor of major bleeding, stroke and mortality in the ARISTOTLE trial (the Apixaban for Reduction in Stroke and Other Thromboembolic Events in Atrial Fibrillation) and RE-LY (Randomized Evaluation of Long-Term Anticoagulant Therapy), and is one of the strongest prognostic factors in the ABC (age, biomarkers, comorbidities) score, the new score for assessing risk of AF patients [22, 23].

The diagnosis of HF (especially HFpEF) in patients with AF remains a clinical challenge, since signs and symptoms, echocardiographic abnormalities and elevated NT-proBNP levels can be caused by both AF alone and by AF with concomitant HFpEF. Since GDF-15 has previously been shown to have diagnostic utility in HFpEF, with similarly or even more elevated levels as compared with patients with HFrEF, and seems to be less influenced by concomitant AF in patients with HF as shown in the present study, it could perhaps be a suitable companion marker next to NT-proBNP to diagnose the presence or absence of HFpEF in patients presenting with AF [9, 24, 25]. This novel hypothesis should be further explored by studying the potential usefulness of GDF-15 and its clinical consequences. The combination of GDF-15 and NT-proBNP to distinguish AF versus HF by additionally comparing levels in patients with AF without HF, as well as by comparing levels in patients with AF before and after cardioversion needs further investigation.

In clinical practice, NP levels are often used for therapy guidance, but these levels can fluctuate in patients with HF and paroxysmal AF, depending on whether they are in SR or AF at the time of measurement [26, 27]. Since NT-proBNP is mainly produced and secreted by the cardiomyocytes in the atria and ventricles in response to haemodynamic wall stress, this marker is known to be sensitive to heart rate and rhythm disturbances [5, 7].

As described previously, most contemporary clinical HF trials use different NP thresholds for the inclusion of patients with and without AF [28–30]. These higher thresholds in patients with AF increase the probability that these patients have actual underlying HF, instead of including patients who have merely AF—a distinction that is especially challenging in patients with HFpEF and AF [31]. For patients with a history of AF but who are in SR at time of blood collection, it is often unclear which threshold to use. This study shows for the first time that patients with a history of AF but who have SR at the time of measurement, have NT-proBNP levels that are much lower and more similar to those patients who have never had AF before, as compared to patients who have AF at time of measurement. Using a higher NP threshold for patients in SR but with a history of AF could result in inappropriately high screen failure rates in clinical trials. In these clinical HF trials, GDF-15 might be considered as an additional marker to distinguish severity of HF apart from AF.

### Limitations

Limitations of this study include the post hoc design. There was a lack of information about the duration between the last episode of AF and screening and number/type of AF episodes the patient had experienced before. Asymptomatic patients with paroxysmal AF could have been missed and regarded as SR patients. Furthermore, no echocardiography data apart from LVEF was available. The NT-proBNP cutoff for inclusion in the BIOSTAT-CHF index cohort could have potentially led to higher inclusion rates of SR patients with more severe HF as compared with those with AF, and as compared with patients included in the validation cohort. However, even though this inclusion criterion differed, similar biomarker patterns were observed in both the index and validation cohort, which is a strength of the present study. Unfortunately, we were not able to further stratify the index and validation cohort in HFrEF and HFpEF, since this would have importantly limited the number of patients in the three rhythm groups within these three HF subtypes.

## Conclusion

The plasma levels of NT-proBNP in HF patients were significantly influenced by the presence of AF at time of measurement, whereas the plasma levels of GDF-15 were independent of underlying AF. Therefore, GDF-15 might have additive value combined with NT-proBNP in the assessment of patients with HF and concomitant AF.

## Electronic supplementary material

Below is the link to the electronic supplementary material.

Supplementary material 1 (DOCX 92 kb)

## Abbreviations

AF: Atrial fibrillation
BIOSTAT-CHF: The BIOlogy Study to Tailored Treatment in Chronic Heart Failure
ECG: Electrocardiogram
GDF-15: Growth differentiation factor-15
HF: Heart failure
LVEF: Left ventricular ejection fraction
NT-proBNP: N-terminal pro-B-type natriuretic peptide
SR: Sinus rhythm
