# Correction to: The influence of atrial fibrillation on the levels of NT-proBNP versus GDF-15 in patients with heart failure

**DOI:** 10.1007/s00392-020-01762-2

**Published:** 2020-11-03

**Authors:** Bernadet T. Santema, Michelle M. Y. Chan, Jasper Tromp, Martin Dokter, Haye H. van der Wal, Johanna E. Emmens, Janny Takens, Nilesh J. Samani, Leong L. Ng, Chim C. Lang, Peter van der Meer, Jozine M. ter Maaten, Kevin Damman, Kenneth Dickstein, John G. Cleland, Faiez Zannad, Stefan D. Anker, Marco Metra, Pim van der Harst, Rudolf A. de Boer, Dirk J. van Veldhuisen, Michiel Rienstra, Carolyn S. P. Lam, Adriaan A. Voors

**Affiliations:** 1 Department of Cardiology, University of Groningen, University Medical Centre Groningen, Hanzeplein 1, 9713 GZ Groningen, The Netherlands; 2 Department of Cardiology, National Heart Centre Singapore, Singapore Duke-NUS Graduate Medical School, Singapore, Singapore; 3 Department of Cardiovascular Sciences, University of Leicester, Groby Road, Leicester, LE3 9QP UK; 4 NIHR Leicester Biomedical Research Centre, Glenfield Hospital, Groby Road, Leicester, LE3 9QP UK; 5 Division of Molecular and Clinical Medicine, School of Medicine University of Dundee, Ninewells Hospital and Medical School, Dundee, DD1 9SY UK; 6 University of Bergen, Bergen, Norway; 7 Stavanger University Hospital, Stavanger, Norway; 8 National Heart & Lung Institute, Royal Brompton & Harefield Hospitals, Imperial College, Sydney St, Chelsea, London, SW3 6NP UK; 9 Robertson Institute of Biostatistics and Clinical Trials Unit, University of Glasgow, University Avenue, Glasgow, G12 8QQ UK; 10 Inserm CIC 1433, Université de Lorrain, CHU de Nancy, Nancy, France; 11 Department of Cardiology (CVK), and Berlin Institute of Health Center for Regenerative Therapies (BCRT), German Centre for Cardiovascular Research (DZHK) partner site Berlin, Charité Universitätsmedizin, Berlin, Germany; 12 Department of Medical and Surgical Specialties, Radiological Sciences and Public Health, Institute of Cardiology, University of Brescia, Brescia, Italy

## Correction to: Clinical Research in Cardiology (2020) 109:331–338 10.1007/s00392-019-01513-y

The original version of this article unfortunately contained a mistake. Section “Results”, second paragraph, first sentence should read:

The plasma levels of NT-proBNP were significantly higher in patients who had AF at baseline, with a median of 3417 pg/mL (1897–6486), as compared with patients who were in SR at baseline; both those who had a history of AF (1788 pg/mL [682–3870], *p *< 0.001) and those who never had AF before (2231 pg/mL [902–5270], *p *< 0.001) (Table 1), also after multivariable adjustment (Table 2).

