# Supplementary material for: The influence of atrial fibrillation on the levels of NT-proBNP versus GDF-15 in patients with heart failure
Source: Clin Res Cardiol. 2019 Jul 1;109(3):331–8. doi: 10.1007/s00392-019-01513-y (PMC7042190; doi:10.1007/s00392-019-01513-y)
Supplement: Supplementary file 1 — Supplementary material 1 (DOCX 92 kb) [file 392_2019_1513_MOESM1_ESM.docx]

**Supplementary Figure 1.** Flowchart of selected patients of the BIOSTAT-CHF index cohort


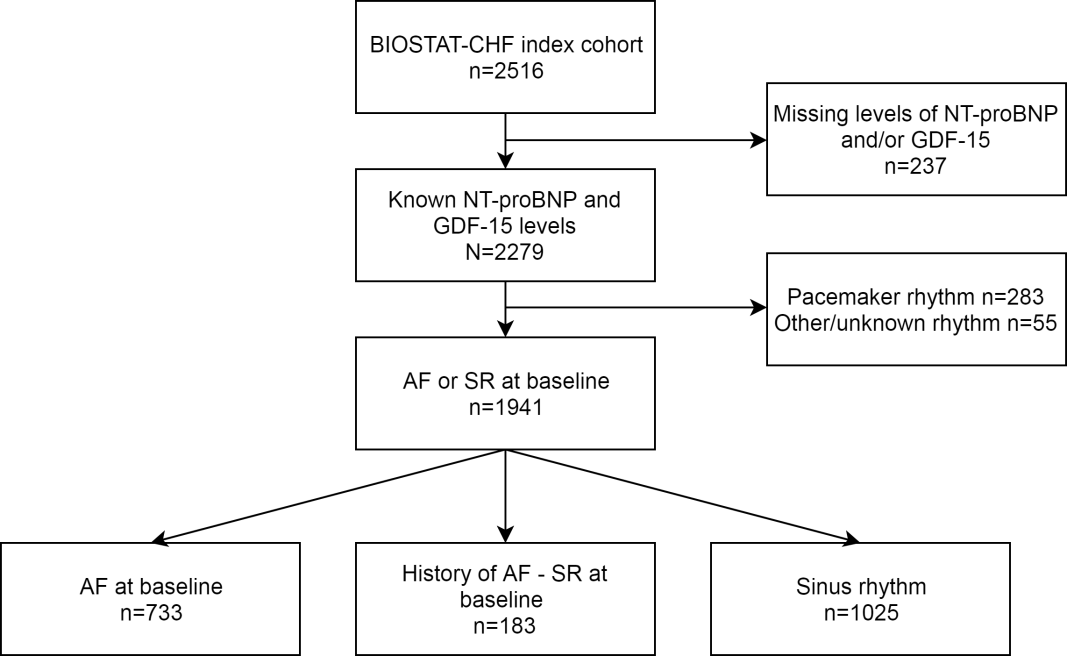


BIOSTAT-CHF=The BIOlogy Study to Tailored Treatment in Chronic Heart Failure, NT-proBNP=N-terminal pro-B-type natriuretic peptide, GDF15=growth differentiation factor 15, AF=atrial fibrillation, SR=sinus rhythm.

**Supplementary Table 1.** Baseline characteristics of the validation cohort, stratified by heart rhythm.

| Clinical characteristic | AF at baseline  N=536 (37%) | History of AF –  SR at baseline  N=156 (11%) | Sinus rhythm N=754 (52%) | P for trend |
| --- | --- | --- | --- | --- |
| Age (years) | 75 ± 10 | 72 ± 11 | 72 ± 11 | <0.001 |
| Women (%) | 169 (32) | 54 (35) | 291 (39) | 0.032 |
| BMI (kg/m2) | 29.3 ± 6.2 | 29.2 ± 6.6 | 28.8 ± 6.4 | 0.347 |
| NYHA (%) |  |  |  | 0.082 |
| I/II | 202(38) | 68(44) | 331(44) |  |
| III | 248 (46) | 70 (45) | 325 (43) |  |
| IV | 86 (16) | 18 (12) | 97 (13) |  |
| LVEF, % | 43 ± 13 | 39 ± 14 | 41 ± 13 | 0.004 |
| Systolic blood pressure (mm Hg) | 125 ± 21 | 127 ± 23 | 127 ± 23 | 0.199 |
| Diastolic blood pressure (mm Hg) | 72 ± 15 | 68 ± 13 | 68 ± 12 | <0.001 |
| Heart rate (beats/minute) | 86 ± 26 | 72 ± 18 | 72 ± 18 | <0.001 |
| History of (%) |  |  |  |  |
| Myocardial infarction | 206 (38) | 76 (49) | 420 (56) | <0.001 |
| Stroke | 117 (22) | 34 (22) | 105 (14) | <0.001 |
| Hypertension | 320 (60) | 94 (61) | 433 (58) | 0.601 |
| Diabetes mellitus | 177 (33) | 62 (40) | 227 (30) | 0.054 |
| COPD | 91 (17) | 38 (25) | 135 (18) | 0.108 |
| Medication (%) |  |  |  |  |
| ACE-inhibitors/ARBs | 362 (68) | 112 (72) | 541 (72) | 0.238 |
| Beta-blockers | 386 (72) | 118 (76) | 541 (72) | 0.605 |
| Loop diuretics | 524 (98) | 153 (98) | 749 (99) | 0.048 |
| Amiodarone | 12 (2) | 19 (12) | 27 (4) | <0.001 |
| Digoxin | 201 (38) | 39 (25) | 13 (2) | <0.001 |
| Verapamil/diltiazem | 17 (3) | 6 (4) | 13 (2) | 0.133 |
| Class 1c antiarrhythmic drugs | 0 (0) | 1 (1) | 0 (0) | 0.016 |
| Ivabradine | 3 (1) | 1 (1) | 33 (4) | <0.001 |
| Laboratory data |  |  |  |  |
| eGFR | 58.5 ± 20.7 | 60.8 ± 23.3 | 62.1 ± 23.3 | 0.017 |
| NT-proBNP (pg/mL) | 2105 [1015, 4472] | 1063 [440, 4094] | 874 [314, 2758] | <0.001 |
| GDF-15 (pg/mL) | 3054 [2000, 5015] | 2787 [1883, 4819] | 2611 [1675, 4212] | <0.001 |

AF=atrial fibrillation, SR=sinus rhythm, BMI=body mass index, NYHA=New York Heart Association, LVEF=left ventricular ejection fraction, COPD=chronic obstructive pulmonary disease, ACE=angiotensin converting enzyme, ARBs=angiotensin receptor blockers, eGFR=estimated glomerular filtration rate, NT-proBNP=N-terminal pro-B-type natriuretic peptide, GDF15=growth differentiation factor 15.
